# The Use of Cannabinoids in Pediatric Palliative Care—A Retrospective Single-Center Analysis

**DOI:** 10.3390/children11020234

**Published:** 2024-02-11

**Authors:** David Tagsold, Irmgard Toni, Regina Trollmann, Joachim Woelfle, Chara Gravou-Apostolatou

**Affiliations:** Department of Pediatrics and Adolescent Medicine, Friedrich-Alexander-Universität Erlangen-Nürnberg, 91054 Erlangen, Germany; davidzk@t-online.de (D.T.);

**Keywords:** cannabinoids, pediatric, palliative care

## Abstract

This data analysis aimed to systematically analyze a pediatric patient population with a life-limiting disease who were administered cannabinoids. It was a retrospective single-center analysis of patients under supervision of the specialized outpatient pediatric palliative care (SOPPC) team at the Department of Pediatrics and Adolescent Medicine of the Friedrich-Alexander-Universität Erlangen-Nürnberg (FAU). Thirty-one patients with a primary diagnosis of neuropediatric, oncologic, metabolic, and cardiologic categories were included. The indications we identified were spasticity, pain, restlessness, anxiety, loss of appetite, epilepsy, and paresis. Certain aspects of quality of life were improved for 20 of 31 patients (64.5%). For nine patients (29%), no improvement was detected. No conclusions could be drawn for two patients (6.5%). Adverse events were reported for six of the thirty-one patients (19.4%). These were graded as mild, including symptoms such as restlessness, nausea, and behavioral issues. We detected no clinically relevant interactions with other medications. We collected fundamental data on the use of cannabinoids by pediatric palliative patients. Cannabinoids are now frequently administered in pediatric palliative care. They seem to be safe to use and should be considered an add-on therapy for other drug regimens.

## 1. Introduction

The administration of cannabinoids to pediatric palliative care patients is increasingly coming into the focus of attention for European treating physicians, patients, and patients’ parents due to the current discussion in Europe, especially in Germany, about the controlled dispensing of cannabis to adults.

The endocannabinoid system (ECS) plays an essential regulatory role in many physiological processes [1] since the ECS has widespread effects on human physiology. Research conducted by Adam Stasiulewicz and colleagues found that the ECS has a regulatory effect, especially in regard to pain management, mood control, fat and glucose metabolism, appetite, immune responses, neuron formation, and protection, as well as inflammation processes [2]. Currently, there are only a few studies on the use of cannabinoids in pediatric palliative care. Individual studies conducted on children and adolescents indicated desirable effects on nausea and vomiting after chemotherapy and on dystonia and spasticity [3,4]. Several studies, as well as three important phase-three randomized controlled trials, showed conclusive evidence for the treatment of therapy-refractory epilepsy with Cannabidiol (CBD) [5]. A recent Canadian study involving 21 pediatric palliative patients described evidence of a desirable effect on pain management [6]. For cannabinoid drugs, evidence-based recommendations for the treatment of children and adolescents are still lacking. Therefore, a scientifically valid consideration of off-label treatment must be based on empirical data and case reports.

In recent years, there has been an increasing use of cannabinoids in Germany for therapeutic purposes among patients similar to those in our study. However, at the time of publication, no standardized guidelines for pediatric use have been established.

Due to the lack of child-centered dosage guidelines issued by drug manufacturers, it is common practice to phase in a drug until the desired symptom control is achieved. For Sativex^®^, the dosing schedule described in the adult summary of product characteristics (SmPC) was used. The recommended application is buccal, with one burst of the spray of an oily drug. The amount of Tetrahydrocannabinol (THC) and CBD administered per spray burst is 2.7 mg of THC and 2.5 mg of CBD. One spray burst is administered daily on the first and second days of treatment (0-0-1). On the third and fourth days of treatment, two bursts each (0-0-2) are recommended. A 15 min interval between two spray bursts is advised. Starting on the fifth day of treatment, the dose can be gradually increased by one burst per day up to a maximum of 12 bursts per day.

Epidyolex^®^ is administered, starting in the first week, with 2.5 mg of CBD/kilogram bodyweight (kgbw) divided into two doses. In the second week, this dosage is increased, according to the dosing schedule described in the SmPC, to 5 mg of CBD/kgbw/day. In the third week, this dosage is increased to 5 mg of CBD/kgbw administered twice daily (10 mg CBD/kgbw/day). Depending on tolerability, the dosage may be increased until desirable symptom control is achieved or up to 10 mg of CBD/kgbw twice daily (20 mg CBD/kgbw/day).

According to a previous publication [7], dronabinol was administered following a dosage regimen individually designed for each patient by the SOPPC team of the FAU. The dosage was increased based on desired symptom control and tolerability.

Cannabis flower dosing differs depending on the route of administration (tea preparation; vaporized). For tea preparation, the daily dose used was 0.5 g of the buds. These were boiled in 500 mL of water. Three times a day, a 150 mL dosage was administered. The patients who vaporized the substance were given a weekly dose of 5 g. It is essential to note the possible influence of different qualities and compositions of the products from different manufacturers, as such differences can have an impact on the therapeutic regime. Using standardized medication, we did not observe any changes in the product or the administration of the product during the treatment of the patients.

This retrospective data analysis aims to systematically analyze a pediatric patient population of 31 patients with life-limiting diseases who were administered a cannabinoid treatment regimen by the SOPPC team of the Pediatric Hospital of the FAU. It investigates the clinical entities in pediatric palliative care for which the pediatricians of the pediatric hospital administered cannabinoids; which medications they administered; which therapeutic goals they targeted with the administration; and how the treating physicians judged the overall efficacy of the treatment. In addition, this study analyzes whether there is evidence for a clinical benefit of this therapy, whether adverse drug reactions occur frequently, and, if so, how they affect the treatment.

## 2. Materials and Methods

This was a retrospective, single-center study conducted at the Department of Pediatrics and Adolescent Medicine of the FAU using patient data from the SOPPC team and the Social Pediatric Center.

The SOPPC team cared for 546 patients between 1 May 2011 and 15 January 2021. We identified all patients who had been treated with cannabinoids within that period, who suffered from a life-limiting disease, and who were under the (co-) care of the SOPPC team and included them in the analysis.

We extracted data from patient records and the hospital’s digital documentation system, documenting home visits and patient contacts in the hospital. These data were anonymized and transferred to an Excel spreadsheet. Among these data, we excluded those regarding patients to whom cannabinoids were administered for less than one week. In one case, a patient refused the administration of Sativex^®^ before the first dose. In another, after Sativex^®^ had been administered twice, the patient refused further application due to the time and effort required to dispense the medication.

From the remaining 31 patients, we collected demographic data; the type of primary diagnosis; the type of cannabinoid that was administered and its indication; the route of administration; whether the drug was switched or discontinued and, if so, why; adverse drug reactions; and whether the desired symptom control was achieved. In addition, we examined the prescribed concomitant medication when the cannabinoid was first administered, whether the therapeutic goal was achieved, and whether the treating physicians assessed overall efficacy. Furthermore, we examined anthropometric data, whether there was an improvement in aspects of daily structure and the duration until desired symptom control was achieved.

Treating physicians discussed the therapeutic goal of the cannabinoid prescription with patients and their parents at the beginning of each treatment. In the context of this study, the treating physicians completed a yes/no questionnaire to quantify whether the desired therapy goal was achieved.

To represent the overall efficacy, two treating physicians independently rated the effectiveness of the therapy using a 3-point Likert scale, ranging from no effect to moderate effectiveness and good effectiveness. To evaluate the agreement between the two treating physicians, we calculated Cohens Kappa.

Due to the multimorbidity of many patients, additional symptoms expected to be influenced by cannabinoids presented alongside the primary indication. These symptoms included pain, spasticity, loss of appetite, depressed mood, restlessness, and anxiety and were determined individually by the treating physicians. Multiple responses were possible.

We analyzed the patients’ contact logs, which were generated for each contact at home or in the clinic, to determine whether there was an impact on the aspects of daily structure. We considered it as an improvement in aspects of daily structure if the patient, parents, or palliative team mentioned that since the start of cannabinoid administration, sleep rhythm had improved, there had been a lower number of seizures, food intake had improved, there was better handling during the day, or similar changes had occurred. Adverse drug reactions were recorded as events that could be linked to the administration of the drug by the patients, parents, or the palliative care team and interfered with the goal of treatment or represented a worsening of the general condition.

Many patients lack communicative ability and therefore are unable to quantify improvements in symptoms such as pain or spasticity. This necessitates gradual dosing and exploratory dose discovery based on observation. The duration until desired symptom control had been achieved was used to quantify this process.

We assessed concomitant medication at the start of cannabinoid administration and included a maximum of three medications in the analysis. Priority was given to medications also affecting the range of symptoms thought to be influenced by the cannabinoid in question. These included opioids, nonopioid analgesics, benzodiazepines, antiemetics, antiepileptics, and muscle relaxants.

For some patients, care was interrupted during the observation period. Reasons for this included overcoming an acute crisis and thus no longer needing care from the SOPPC team as well as receiving care from a different SOPPC team after relocation.

Statistical analysis was performed using the IBM SPSS Statistics 28 program and, in the other case, using pivot tables.

The corresponding ethics commission of the FAU approved this study through consultation according to §15 Berufsordnung für Ärzte in Bayern, with the application number: 222:21 BC due to the retrospective study design and total anonymization.

## 3. Results

From 1 May 2011 and 15 January 2021, we included 31 patients in the analysis. Following the amendment of cannabis legislation in Germany in March 2017, enabling the coverage of cannabis expenses through health insurance, six patients underwent cannabinoid treatment since 2011. The remaining 25 patients were treated, after the 2017 law change, until 2021. Sixteen were female, and fifteen were male. Their median age at the time of initial medication administration was 10.0 years (IQR 7.0–18.0), with the youngest treated being 1 year old and the oldest being 33 years old. (Table 1)

**Figure 1:**
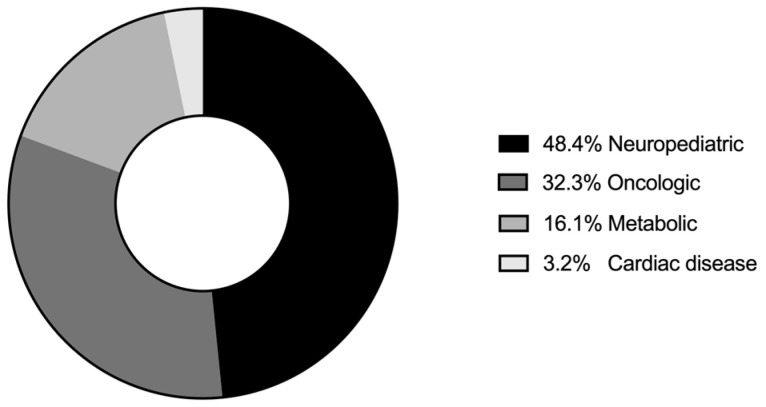
Diagnostic categories of primary diagnosis.

The categories of the primary diagnosis comprised fifteen (48.4%) neuropediatric patients, ten (32.3%) oncologic patients, five (16.1%) metabolic patients, and one (3.2%) patient with cardiac disease. (Figure 1)

Prescribed medications included dronabinol (THC; *n* = 20; 64.5%), Sativex^®^ (THC + CBD; *n* = 8; 25.8%), cannabis buds (THC + CBD; *n* = 2; 6.5%), and Epidyolex^®^ (CBD; *n* = 1; 3.2%), as depicted in Table 2.

### 3.1. Attempted Symptom Control

The primary indications for the administration of the cannabinoid medications were spasticity (*n* = 11), pain (*n* = 8), restlessness (*n* = 5), loss of appetite (*n* = 3), anxiety (*n* = 2), epilepsy (*n* = 1), and paresis (*n* = 1).

An analysis of the indications for the use of a cannabinoid according to the underlying condition revealed that of the fifteen patients who had an underlying neuropediatric condition, seven (46.7%) received the medication due to spasticity, three (20.0%) were administered the medication due to restlessness, two (13.3%) were administered the medication due to pain, and one (6.7%) each received medication due to anxiety, paresis, or epilepsy.

Among the ten oncologic patients, the indications were as follows: five patients (50.0%) received the medication due to pain, two (20.0%) were administered the medication due to loss of appetite, and one patient each (10.0%) was administered medication for spasticity, anxiety, or restlessness.

For three patients (60.0%) who had metabolic disease (*n* = 5), the medication was administered for spasticity, and for one patient each (20.0%), it was administered for pain or restlessness. The patient with cardiac disease took the medication primarily to enhance their appetite.

### 3.2. Choice of Agent for Indication

Dronabinol (THC), administered a total of 20 times, was prescribed for 11 (55.0%) of the patients primarily for spasticity, four times (20.0%) for pain, four times (20.0%) for restlessness, and one time (5.0%) for paresis. Sativex^®^ (THC + CBD), administered to eight patients, was prescribed four times (50.0%) due to pain, three times (37.5%) due to loss of appetite, and one time (12.5%) due to anxiety. Cannabis buds (THC + CBD) were prescribed one time (50.0%) for restlessness and one time (50.0%) for anxiety. Epidyolex^®^ (CBD) was prescribed once (100.0%) for epilepsy.

### 3.3. Overall Efficacy and Therapeutic Goal

**Figure 2:**
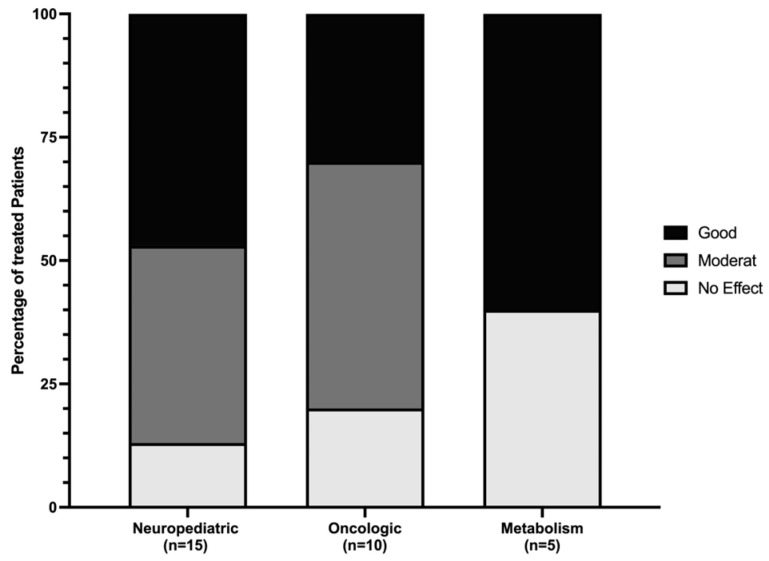
Efficacy related to diagnostic category.

Of the 20 patients treated with dronabinol, the treating physicians reported overall efficacy to be “good” in 45.0% (*n* = 9) and “moderate” in 30.0% (*n* = 6) and to have “no effect” in 25.0% (*n* = 5). For Sativex^®^ (*n* = 8 patients), the treating physicians rated the overall efficacy as being “good” in 12.5% (*n* = 1) and “moderate” in 62.5% (*n* = 5) and as having “no effect” in 25.0% (*n* = 2). For the two patients treated with cannabis buds and the one treated with Epidyolex^®^, the treating physicians rated the overall efficacy as “good” (100.0%). The overall efficacy in relation to the diagnostic categories of the primary diagnosis is depicted in Figure 2. Due to the small number of cardiologic patients, they were not depicted. In the case of the patient with cardiac disease, efficacy was described as “good”.

Treatment with dronabinol led to the achievement of the therapeutic goal in 17 of the 20 cases (85.0%). For Sativex^®^, the goal was reached in five out of eight cases (62.5%). In the cases in which cannabis buds and Epidyolex^®^ were administered, the therapeutic goal was achieved in all cases (100.0%).

For 90.9% (*n* = 10) of the patients for whom the medication was indicated for the control of spasticity (*n* = 11), the treating physicians rated the overall efficacy as “good” (*n* = 5; 45.5%) or “moderate” (*n* = 5; 45.5%). We observed no effect for one patient (*n* = 1; 9.1%). For 75.0% (*n* = 6) of the patients for whom the treatment of pain was the main focus (*n* = 8), the treating physicians rated the overall efficacy as “good” (*n* = 3; 37.5%) or “moderate” (*n* = 3; 37.5%). We observed no effect for two patients (*n* = 2; 25.0%). We detected no effect in 60.0% (*n* = 3) of the patients who were administered a cannabinoid medication due to restlessness (*n* = 5). For two patients, the effect was rated as “good” (*n* = 2; 40.0%). For three patients, the medication was prescribed to increase appetite. In two patients, the effect was considered “moderate” (*n* = 2; 66.7%). For one patient, we detected no effect (33.3%). For two patients, who received the medication for anxiety, the effect was rated as “good” once (50.0%) and “moderate” once (50.0%). For the patients to whom the medication was prescribed to treat paresis (*n* = 1; 100.0%) or epilepsy (*n* = 1; 100.0%), the effect was rated as “good” both times. The calculated linear weighted Cohen’s Kappa for the inter-rater reliability of the treatment effectiveness ratings between the two treating physicians was 0.744.

**Figure 3:**
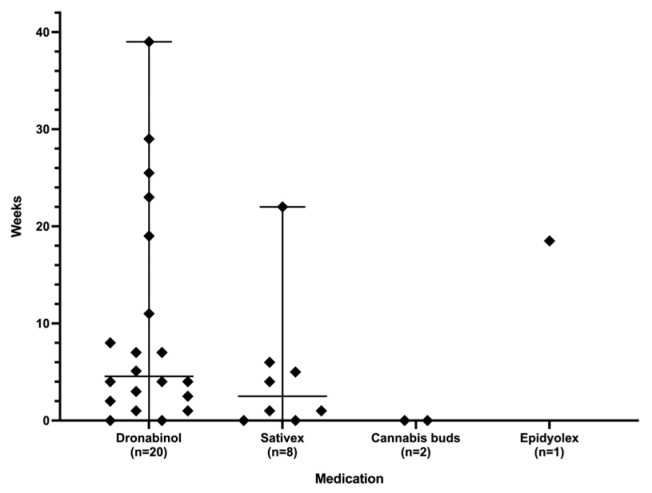
Duration until achievement of desired symptom control.

The time intervals required to attain the desired symptom control are depicted in Figure 3. The median duration was 4.6 weeks for dronabinol, 2.5 weeks for Sativex^®^, immediate for cannabis buds, and 20 weeks for the patient using Epidyolex^®^.

### 3.4. Aspects of Daily Structure

The aspects of daily structure improved in 64.5% (*n* = 20) of the 31 patients via administering cannabinoid medication, whereas no improvement could be observed in 29.0% (*n* = 9) of the patients. No statement about improvement could be made for two patients (6.5%). Upon conducting a more detailed analysis of the results, it was determined that 86.6% (*n* = 13) of the neuropediatric patients benefited from the medication. For the oncologic patients, this value was 40.0% (*n* = 4), corresponding to 40.0% (*n* = 2) for patients with metabolic disease. The patient with cardiac disease exhibited no improvements in his aspects of daily structure.

### 3.5. Administration

While dronabinol (THC) and Epidyolex^®^ (CBD) were administered as an oil-based solution (*n* = 21; 67.7%) and Sativex^®^ (THC/CBD) was applied as a spray (*n* = 8; 25.8%), cannabis buds (CBD/THC) were either used as a tea (*n* = 1; 3.2%) or vaporized (*n* = 1; 3.2%).

Thirteen of the fifteen (86.7%) neuropediatric patients were administered dronabinol (THC), one (6.7%) used cannabis buds (CBD/THC), and one was administered (6.7%) Epidyolex^®^ (CBD). Seven of the ten (70.0%) oncologic patients were administered Sativex^®^ (CBD/THC), while two (20.0%) and one (10.0%) were administered dronabinol (THC) and cannabis buds (CBD/THC), respectively. Five (100.0%) of the metabolic patients received dronabinol (THC). The cardiologic patient was administered Sativex^®^ (CBD/THC).

### 3.6. Symptom Control

In addition to the primary indication, many multi-morbidity patients had other symptoms for which a beneficial change was expected. Overall, the medications were used to control pain in 16 patients and spasticity in 14 patients. Additionally, restlessness was sought to be improved in 13 patients. We expected the seizure frequency and severity to improve in 10 patients.

### 3.7. Adverse Drug Reactions

We observed adverse drug reactions in 25.0% (*n* = 5) of the patients treated with dronabinol (*n* = 20). All these patients had been undergoing treatment for a neuropediatric disease. The reactions comprised sweating, nausea, anxiety, behavioral abnormalities, and psychiatric symptoms. Among them, 40.0% (*n* = 2) were female, and 60.0% (*n* = 3) were male. Adverse drug reactions also occurred in 12.5% (*n* = 1) of the patients treated with Sativex^®^ (*n* = 8). In this case, the reaction manifested in the form of fatigue. This patient was treated because of an oncologic disease. The two patients treated with cannabis buds and the one patient treated with Epidyolex^®^ reported no adverse drug reactions.

### 3.8. Discontinuation

Treatment was discontinued prematurely in 29.0% (*n* = 9) of the total 31 cases. It was discontinued for 30.0% (*n* = 6) of those treated with dronabinol (*n* = 20) and for 37.5% (*n* = 3) of those treated with Sativex^®^ (*n* = 8). The reasons for the discontinuation of the dronabinol treatment were lack of efficacy (*n* = 4), a suspected adverse drug reaction (*n* = 1), and the request of the treating physicians for a pain consultation and the corresponding discontinuation of pain medication required for this (*n* = 1). In the cases treated with Sativex^®^, the reasons were lack of efficacy (*n* = 1) and the effort required to administer the medication (*n* = 2). The remaining 22 patients (70.9%) did not discontinue treatment during the reference period.

Care provided by the SOPPC was interrupted for 32.3% (*n* = 10) of the patients during the observation period. In total, 38.7% (*n* = 12) of the patients died during this period.

### 3.9. Concomitant Medication

The median number for concomitant medications also targeting the range of symptoms thought to be influenced by the cannabinoid was two (range: 0–3). Among the patients taking concomitant anti-seizure medications (ASMs), 18 took a median of two ASMs (range 2). Eight patients taking concomitant non-opioid analgesics had a median of one analgesic. Eight patients were treated with one opioid (III), and five patients were treated with one muscle relaxant each.

## 4. Discussion

In this study, cannabinoids were used for several indications, including spasticity, pain, restlessness, loss of appetite, agitation, anxiety, and paresis. The indications for the administration of a medication containing cannabinoids are in agreement with previous data [8,9,10,11,12,13]. We expected a positive development of the respective constellation of symptoms based on specific publications dealing with the administration of cannabinoids in the pediatric palliative setting [3,4,6].

Upon examining a putative improvement in aspects of daily structure, we found that patients with neurological diseases benefited the most from the medication. This can be related to the fact that these patients received the medication primarily due to symptoms of spasticity. Previous publications showed that cannabinoids had a therapeutic effect on treating refractory spasticity [4,5,14], a finding we could confirm in our cohort. The therapeutic goal was achieved in the majority of cases, so it seems to be safe to add cannabinoid medication to the existing therapeutic regimen to further improve specific symptoms. Studies have yet to show if they can replace other medications. Due to the multi-morbidity of many patients, a wide range of medications was used. We did not detect interactions and resulting adverse drug reactions.

Currently, there are no child-friendly methods of application for cannabinoids available. Sativex^®^ was discontinued mainly due to the time and effort required to dispense the medication, with 15 min between each spray burst, and the bitter taste of the spray. For a more suitable and reproducible use in pediatric palliative care, more child-friendly preparations are needed to ensure a consistent application of the medication.

Cannabinoids can cause adverse drug reactions. Some medications that affect the central nervous system impact the brain’s developmental capabilities [15,16]. There is a distinction between the short-term and long-term effects of cannabinoids. The short-term effects include dizziness, cognitive impairment, impaired free recall, impaired working memory and procedural memory, anxiety, and sedation [5,6,17]. These drug reactions are brief and reversible through abstinence. A small percentage of our patients exhibited some of the short-term effects identified in that study.

The younger the patients to whom cannabinoids are administered, the higher the risk of long-term effects [18], which include triggering psychosis and schizophrenia in individuals predisposed to mental illness [19]. There is a risk of unimpeded development, especially in children, due to medical malpractice in the form of, for example, a non-adapted dosage regimen or drug interactions [20]. Studies are needed so that pediatricians can make clear recommendations.

The chance of withdrawal due to adverse events is significantly higher for patients treated with cannabinoids than for controls. Before treatment with cannabinoids, a detailed evaluation should be conducted with the patient and the parents regarding potential improvements and common adverse events [21]. In our study, only one patient withdrew because of adverse events.

Other phytocannabinoids in the cannabis plant, such as tetrahydrocannabivarin, cannabigerol, and cannabichromene, may have additional effects of therapeutic interest [22]. To date, it remains unclear which part of the plant is most effective for treating a specific symptom. It could also be that a combination of different cannabinoids, a specific route of administration, or a given dosage might show the best effect [23]. Moreover, whether the best dose or combination of cannabinoids for one symptom complex are as effective as they would be for other indications has not been studied [5].

The demand for medical cannabis seems to be increasing, as demonstrated in a study from Australia that shows that since the legalization of medical cannabis in 2016, the number of prescriptions for medical cannabis has increased significantly [24]. Consistent with this trend, our study reflects a similar observation, as the majority of cannabinoid treatments commenced after the legalization of medical cannabis in Germany in 2017.

Due to the small number of patients, i.e., 31, our results cannot be considered nationally representative. Our study also included the treatment of young adults aged 18 to 33. These older patients were treated for a congenital disease by the SOPPC team. It was found that the treatments administered in our study were not influenced by age or developmental factors. For future studies, especially those designed to establish a dosing regimen, it is probably necessary to exclude patients over the age of 18 years. Since dronabinol has been used primarily to treat neurologically severely affected children with limited communication ability, dose finding with this medication highly depends on the child in question’s observation. Symptom control and the effects of medication are difficult to quantify. However, in the assessment of treatment efficacy, the Cohen’s Kappa value of 0.744 indicates a substantial degree of agreement between the two treating physicians [25]. A possible confirmation bias of parents and treating physicians based on expectations cannot be ruled out. Parents who wish for the medication to be effective might interpret their children’s behavior differently than those who doubt the efficacy of cannabinoid medication. More studies are needed in this area, with specialized analyses of symptoms using quantifiable methods. Spasticity could be monitored with the modified Ashworth scale [26]. In addition, it is difficult to monitor the number of days of treatment and compliance with a medication regimen, as there are patients currently not being cared for by the SOPPC team. Most of them are in a phase of their disease that does not require active care. These patients can be readmitted to active care as a crisis intervention. Prospective studies are required to ensure that medication is continuously monitored.

Based on our analysis, it seems to be safe to conclude that cannabinoids can be used safely and effectively in treating neurologic, oncologic, metabolic, and cardiologic pediatric disorders. In this study, we found that cannabinoids were used to relieve spasticity, pain, restlessness, or anxiety; increase appetite; or reduce seizure frequency in cases of treatment-refractory epilepsy. They showed a low spectrum of adverse events, and no obvious drug interactions, which generally must be considered, were found to be attributable to cannabinoid medication. Due to the lack of available data, cannabinoids should not be used as first-line therapy. However, they can be used, especially in the pediatric palliative setting, as an add-on therapy in multi-morbidity and less severely affected children to achieve a quality-of-life improvement. The aspects of daily structure improved in many cases, which is especially important for pediatric palliative patients.

## Figures and Tables

**Table 1:** Cohort description.

	*n*	%
	31	100
**Gender**		
Female (%)	16	51.6%
Male (%)	15	48.4%
**Age**		
<1 year	0	0%
1–3 years	2	6.5%
4–12 years	17	54.8%
13–18 years	5	16.1%
19–24 years	5	16.1%
24–33 years	2	6.5%
**Diagnostic categories**		
Neuropediatric	15	48.4%
Oncologic	10	32.3%
Metabolic	5	16.1%
Cardiologic	1	3.2%
**Attempted symptom control**		
Spasticity	11	35.4%
Pain	8	25.8%
Restlessness	5	16.1%
Loss of appetite	3	9.7%
Anxiety	2	6.5%
Epilepsy	1	3.2%
Paresis	1	3.2%

**Table 2:** Different aspects of cannabinoid medications.

	*n*	%
	31	100
**Prescribed medication**		
Dronabinol (THC)	20	64.5%
Sativex (THC + CBD)	8	25.8%
Cannabis buds (THC + CBD)	2	6.5%
Epidyolex (CBD)	1	3.2%
**Assessment of overall efficacy (Physician)**		
Good	13	41.9%
Moderate	11	35.5%
No effect	7	22.6%
**Discontinuation of medication**		
Yes	10	32.3%
No	21	67.7%
**Reasons for discontinuation**		
Lack of efficacy	5	16.1%
Effort of medication administration	2	6.5%
Physician’s request	1	3.2%
Lack of compliance	1	3.2%
Adverse events	1	3.2%
**Adverse events (multiple answers could be given)**		
Fatigue	1	
Anxiety	1	
Behavioral problems	1	
Sweating	1	

## Data Availability

The data presented in this study are available on request from the corresponding author. The data are not publicly available due to privacy reasons.
